# Uptake and accumulation of emerging contaminants in processing tomato irrigated with tertiary treated wastewater effluent: a pilot-scale study

**DOI:** 10.3389/fpls.2023.1238163

**Published:** 2023-08-23

**Authors:** Michele Denora, Vincenzo Candido, Gennaro Brunetti, Francesco De Mastro, Sapia Murgolo, Cristina De Ceglie, Carlo Salerno, Giuseppe Gatta, Marcella Michela Giuliani, Andi Mehmeti, Ruud P. Bartholomeus, Michele Perniola

**Affiliations:** ^1^ Department of European and Mediterranean Cultures, University of Basilicata, Via Lanera, Matera, Italy; ^2^ Department of Soil, Plant, and Food Science, University of Bari, Bari, Italy; ^3^ Water Research Institute (IRSA), National Research Council (CNR), Bari, Italy; ^4^ Department of Agricultural Sciences, Food, Natural Resources and Engineering (DAFNE), University of Foggia, Foggia, Italy; ^5^ Mediterranean Agronomic Insitute of Bari (CIHEAM Bari), Valenzano, Italy; ^6^ KWR Water Research Institute, Nieuwegein, Netherlands; ^7^ Soil Physics and Land Management, Wageningen University & Research, Wageningen, Netherlands

**Keywords:** emerging contaminants (EC), wastewater irrigation, water reuse, plant uptake, tomato, soil contamination

## Abstract

The reuse of treated wastewater for crop irrigation is vital in water-scarce semi-arid regions. However, concerns arise regarding emerging contaminants (ECs) that persist in treated wastewater and may accumulate in irrigated crops, potentially entering the food chain and the environment. This pilot-scale study conducted in southern Italy focused on tomato plants (*Solanum lycopersicum* L. cv Taylor F1) irrigated with treated wastewater to investigate EC uptake, accumulation, and translocation processes. The experiment spanned from June to September 2021 and involved three irrigation strategies: conventional water (FW), treated wastewater spiked with 10 target contaminants at the European average dose (TWWx1), and tertiary WWTP effluent spiked with the target contaminants at a triple dose (TWWx3). The results showed distinct behavior and distribution of ECs between the TWWx1 and TWWx3 strategies. In the TWWx3 strategy, clarithromycin, carbamazepine, metoprolol, fluconazole, and climbazole exhibited interactions with the soil-plant system, with varying degradation rates, soil accumulation rates, and plant accumulation rates. In contrast, naproxen, ketoprofen, diclofenac, sulfamethoxazole, and trimethoprim showed degradation. These findings imply that some ECs may be actively taken up by plants, potentially introducing them into the food chain and raising concerns for humans and the environment.

## Graphical Abstract

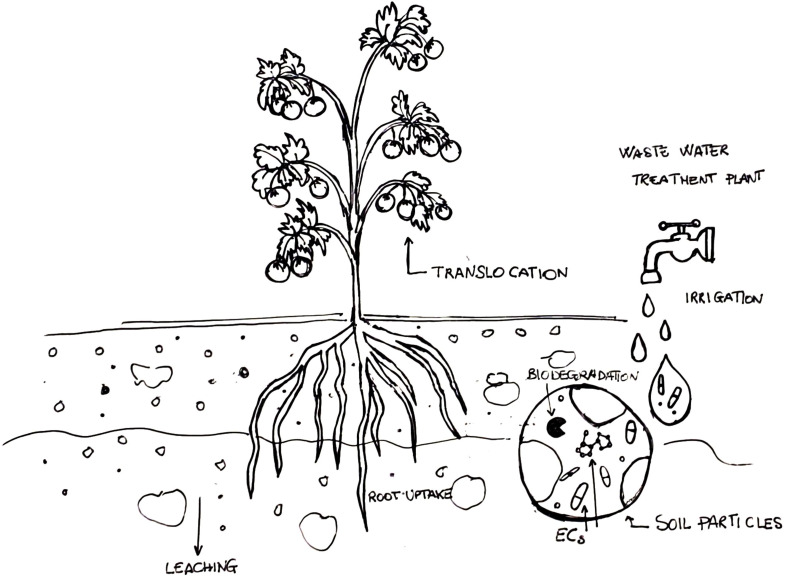

## 1 Introduction

Globally, 70% of freshwater is used for agriculture, with substantially greater figures in developing countries. Agricultural water scarcity will intensify on more than 80% of global croplands (Liu et al., 2022). Meanwhile, population expansion, fast urbanization, and climate change all exacerbate water demand, resource depletion, and water pollution (Boretti and Rosa, 2019). Irrigation management is frequently complicated in water-stressed regions. The economy, crop patterns, output, food demand, and consumption will all be impacted in various ways by climate change and water scarcity (Zingaretti et al., 2013). To ensure water resources’ sustainability, non-conventional water resources are becoming a reality (Chen et al., 2021). Municipal treated wastewater (hereafter referred to as reclaimed water) is increasingly being used in arid and semi-arid regions as a major alternative source of irrigation water (Ungureanu et al., 2020). Irrigation with treated wastewater has long been practiced in the Mediterranean basin, particularly in water-scarce regions where treated wastewater reuse accounts for up to 5-12% of total treated wastewater effluent. By 2021, about 44 nations used daily treated wastewater for agricultural irrigation (Hashem and Qi, 2021). The Middle East and North Africa (15%) and Western Europe (16%) have exceptionally high rates of treated wastewater reuse (Jones et al., 2021).

Reusing treated wastewater for irrigation offers numerous benefits, such as increased profitability for farmers, reduced need for expensive fertilizers due to nutrient-rich water, and preservation of freshwater resources. However, it also poses challenges related to soil salinity, human health risks from pathogens and heavy metals, and social and economic considerations. In recent years, there has been increasing concern about the environmental concerns posed by so-called “emerging contaminants” (Taheran et al., 2018). The ECs are predominantly unregulated anthropogenic chemicals that occur in trace concentrations in air, soil, water, food, and human and animal tissues (Rout et al., 2021). Following uptake into edible plant parts, EOCs may eventually enter in the food chain, with associated human exposure (González García et al., 2019). Irrigation water (Shi et al., 2022), irrigated soils (Rogowska et al., 2020), marketed crops (Ben Mordechay et al., 2021), and even biological samples such as human urine (Schapira et al., 2020) have been found to contain ECs. Once in the soil, the ECs go through several processes that determine their fate: sorption-desorption, transport, biotic and/or abiotic transformation, and plant uptake. Lipophilicity, size, H-bond donors/acceptors moieties, and charge of ECs all influence their sorption attraction to soil particles (Gworek et al., 2021; Strawn, 2021). Soil properties, specifically soil organic matter content, pH, clay content, and clay type, also influence this process (Fu et al., 2016; De Mastro et al., 2022a). Desorption (the return of an adsorbed fraction to the soil solution) is a governing factor, particularly during the rainy season, because rainwater contains negligible concentrations of ECs, altering the EC sorption equilibrium in the soil (Ben Mordechay et al., 2022). While easily degraded ECs are transformed and/or metabolized during wastewater treatment, more persistent ECs remain in the effluents and may accumulate in soils and be taken up by plants (Ben Mordechay et al., 2018).

By implementing appropriate treatment technologies, monitoring soil and water quality, and employing careful irrigation practices, wastewater irrigation can be a safe and effective solution to address water scarcity and promote sustainable agriculture (Mishra et al., 2023). Scientific studies have attempted to characterize the uptake of EC from reclaimed water into different crops such as tomatoes (Christou et al., 2017), strawberries, and lettuce (Hyland et al., 2015; González García et al., 2019; Sunyer-Caldú et al., 2022), some common vegetables such as carrot, radish, spinach, and artichoke (Hussain et al., 2019; Beltrán et al., 2020; De Mastro et al., 2023), and others such as cucumber, eggplant, long bean, and wheat (Liu et al., 2020). The bioaccumulation factor range of ECs is normally rather extensive, depending on the examined plant, exposure length, soil qualities, climate conditions, particularly temperature and humidity, and, most crucially, the molecule’s physicochemical features (Ben Mordechay et al., 2018). Yet, the synergistic effects of multiple contaminants on soil and crops are poorly understood (Lyu et al., 2022).

This study aimed to investigate the occurrence and fate of emerging contaminants (pharmaceuticals) in soil and (*Solanum lycopersicum* L.) tomato plants irrigated with municipal treated wastewater in Southern Italy. A field experiment was designed with tomato plants grown in lysimeters and subjected to freshwater and contaminated wastewater irrigation treatments. The study uses lysimeters in an open field rather than a greenhouse to closely simulate real agricultural settings, yielding insights for extrapolation studies in wastewater-related research. Furthermore, the study adds new realistic evidence on the levels of emerging contaminants in tomatoes grown on soil (lysimeters) media irrigated with fresh and treated wastewater, as well as useful information on the distribution of emerging contaminants tailored to the needs of Mediterranean environments.

## 2 Materials and methods

### 2.1 Experimental design and data collection

**Figure 1:**
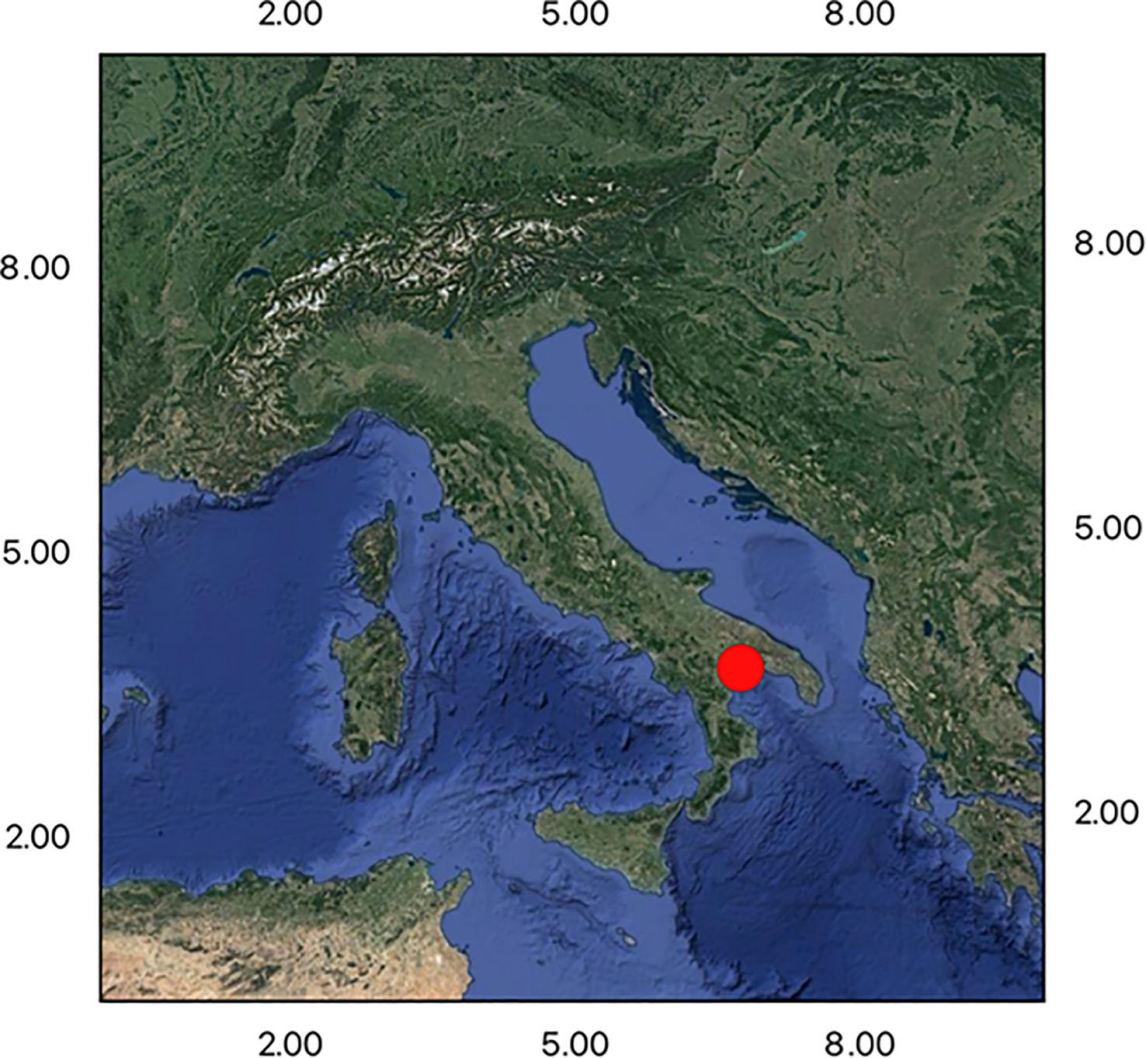
Map of Italy and location of experimental site.

The experimental study (**Figure 1**) was carried out at the ALSIA Metapontum Agrobios Research Center, province of Matera (N 40 23´, E 16 47´), Italy. The climate in the region is Mediterranean, with moderate, wet winters, and hot, dry summers. The average temperature in the summer months (June to September) ranges from 24°C to 28°C, while the average temperature in the winter months (December to February) ranges from 5°C to 11°C. The total annual rainfall averages around 600-700 millimeters, with most of the precipitation occurring from November through April.

**Figure 2:**
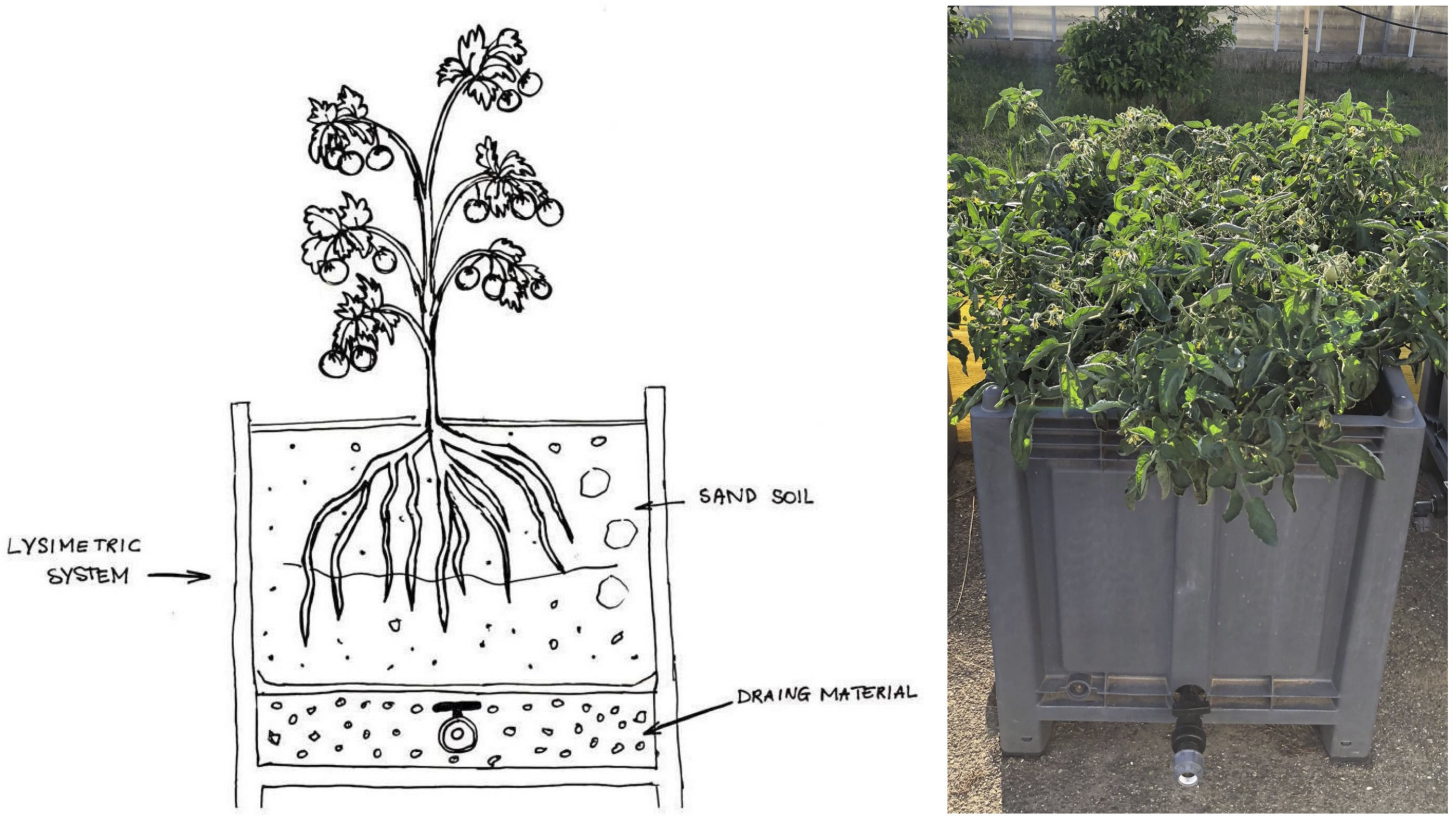
Schematic representation of the lysimetric weighing system, for determining water consumption, water flow, and mass balance of ECs.

On 17/06/2021, the tomato cultivar ‘Taylor F1’ (*Solanum lycopersicum* L.; formerly Lycopersicon esculentum Mill.) was transplanted in weighing lysimeters (**Figure 2**).

**Figure 3:**
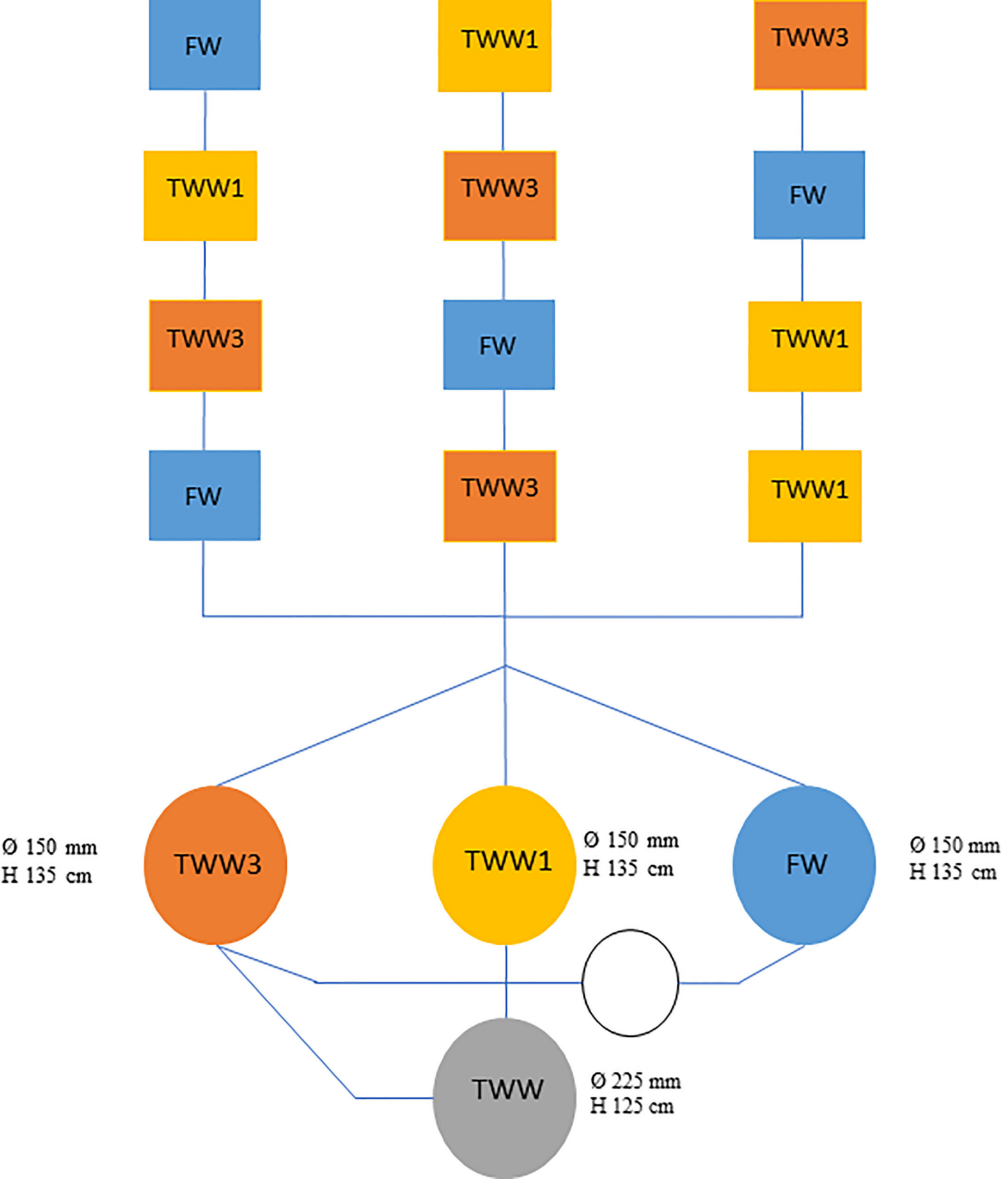
Detail of the experimental scheme (rectangles - tanks in which the treatments were prepared; red - randomized lysimeters; circle - tanks used for the storage of the treated water and its safe disposal).

Pre-cultivated tomato seedlings in 180-hole polystyrene honeycomb containers were transplanted into 0.8 m^3^ tanks at the 3^rd^-4^th^ true leaf stage for each experimental treatment distributed according to the randomized block experimental scheme with four (4) repetitions (**Figure 3**). The experimental design entailed comparing three irrigation treatments:

i) irrigated with surface freshwater (FW) as control, obtained from the irrigation network system that is normally used by the farmers in the area for crop irrigation;
ii) irrigation with tertiary (TWW) municipal wastewater spiked with the addition of target contaminants in a dose comparable to the European average concentration (TWWx1);
iii) irrigation with tertiary (TWW) municipal wastewater spiked with emerging contaminants in a triple dose (TWWx3).

TWW effluent from a standard municipal wastewater treatment plant (WWTP) at the experimental site (Ferrandina, Italy) was utilized to determine TWWx1 and TWWx3 irrigation treatments. Rapid sand filtration (rSF) and UV treatment are used for tertiary treatment and disinfection. The experimental design includes four lysimetric measures (plots) for each irrigation treatment.

Among the ECs were clarithromycin, sulfamethoxazole, trimethoprim, carbamazepine, diclofenac, fluconazole, climbazole, ketoprofene, metoprolol, and naproxen. These substances were specifically chosen due to their prevalence in wastewater; they are often not completely eradicated during standard treatments. **Table 1** lists the chemical structures and attributes of the selected ECs. The concentration of these EC in treated wastewater ranged from low ng L^−1^ to low µg L^−1^ (Ben Mordechay et al., 2021). Standards (> 98% of purity) were used to prepare the multi-compound stock standard solution (1000 ppm). This solution was added to wastewater used for irrigation to achieve the concentration of 200 and 600 μg L^−1^ of each compound and obtain TWWx1 and TWWx3.

**Table 1:** Physicochemical Properties (Mw, Molecular Weight; Water Solubility; KOW, Octanol/Water Coefficient; pKa, Acid Ionization Constant) of the Selected ECs.

ECs	Molecular Weight g mol^-1^	Chemical Structure	Chemical Class	Water Solubility mg L^-1^	KOW	pKa
Clarithromicin	748	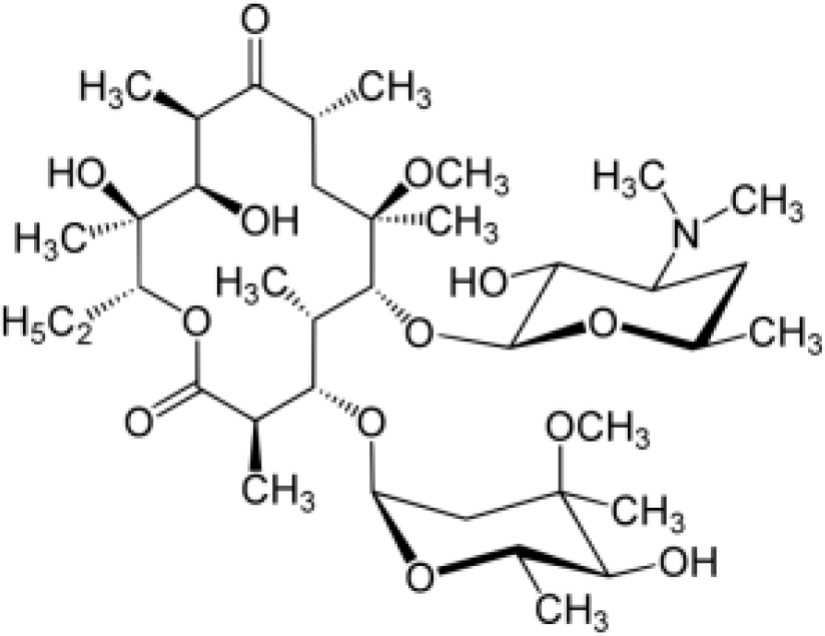	antibiotic	1.693 at 25°C	3.16	8.99
Trimethoprim	290.32	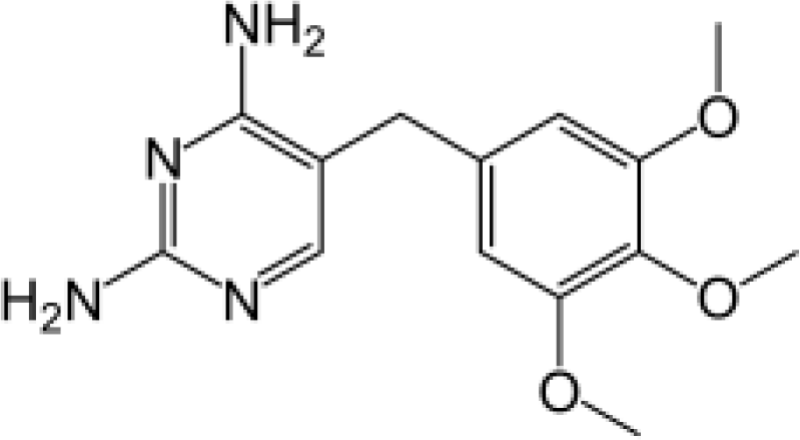	antibiotic	400 at 25°C	0.91	7.12
Sulfamethoxazole	253.28	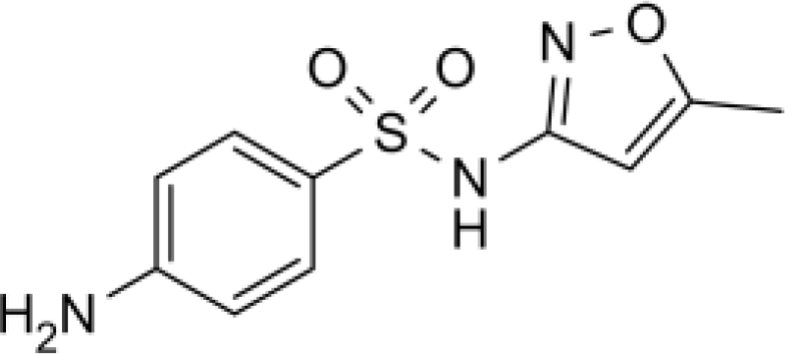	antibiotic	610 at 37°C	0.89	1.6
Fluconazole	306.27	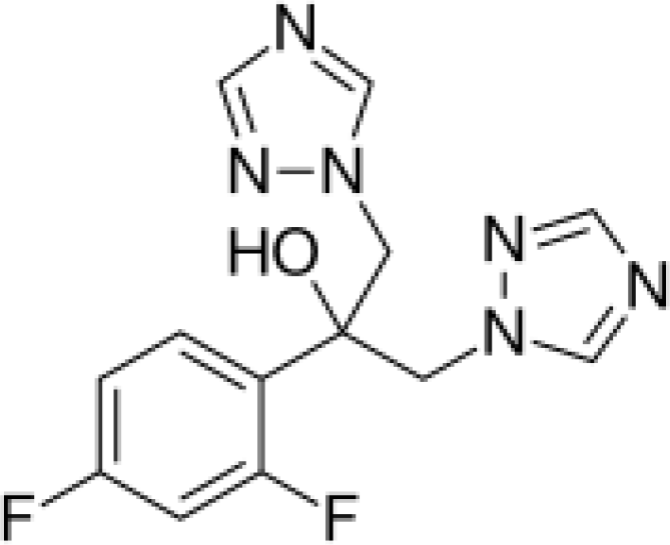	antifungal	4,363 at 25°C	0.25	2.27
Climbazole	292.76	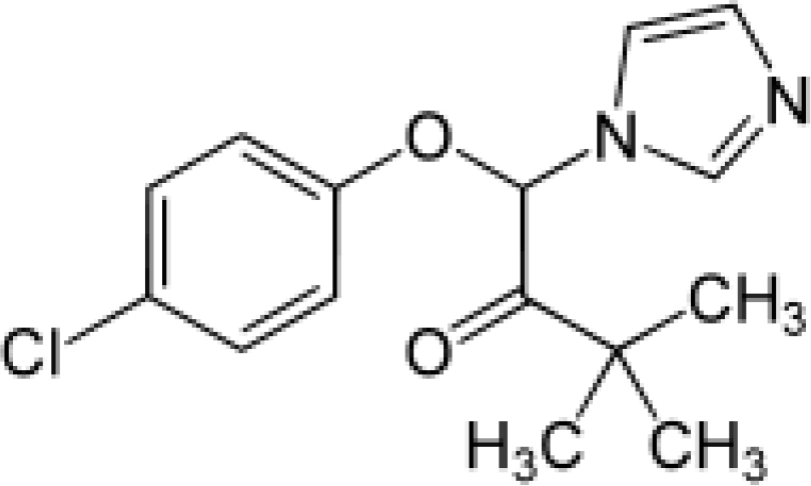	antifungal	58 at 25°C	3.76	6.49
Diclofenac	296.1	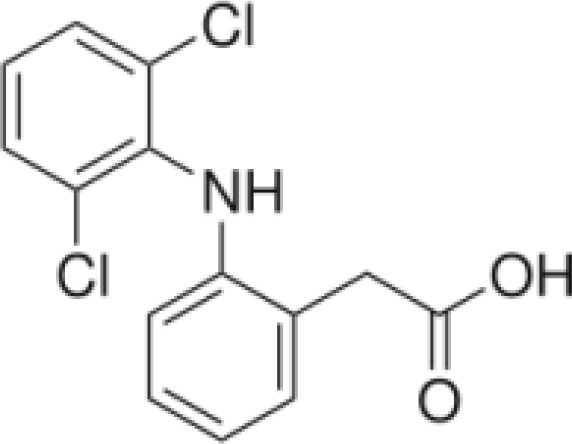	anti-inflammatory	2.37 at 25°C	4.15	4.15
Ketoprofene	254.28	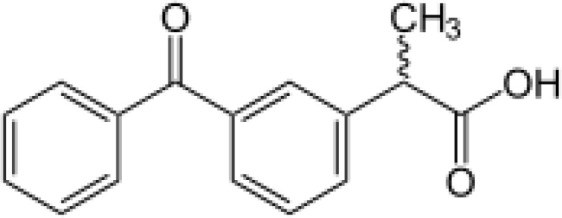	anti-inflammatory	51 at 22°C	3.12	4.45
Naproxen	230.26	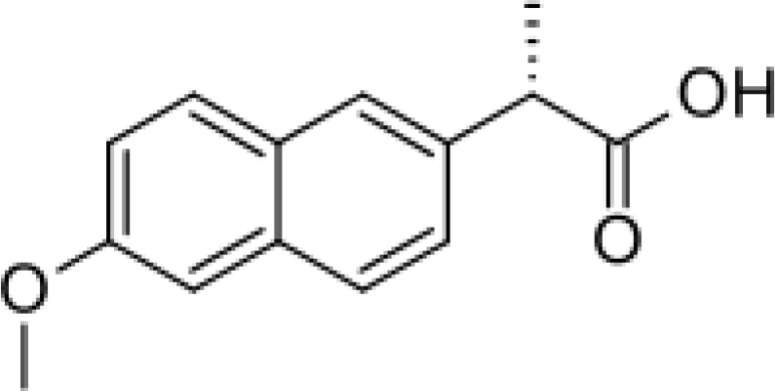	anti-inflammatory	15.9 at 25°C	3.18	4.15
Metoprolol	267.36	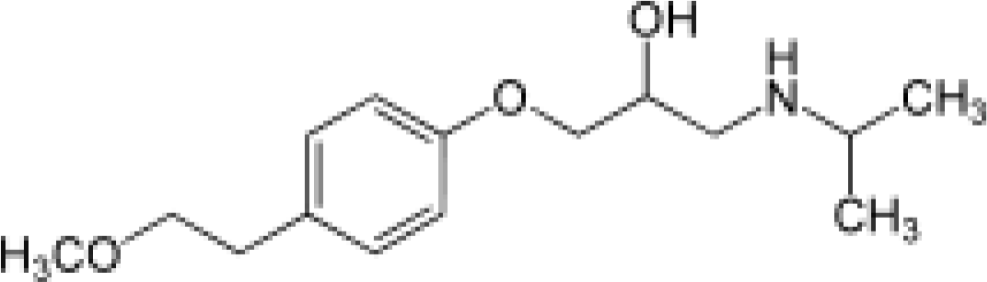	beta blockers	0.4 at 25°C	1.88	9.7
Carbamazepine	236.27	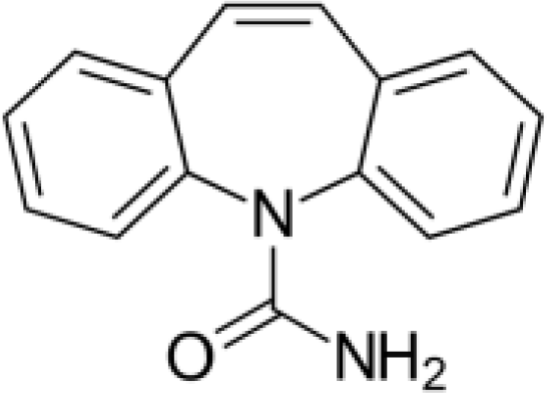	antidepressants	18 at 25°C	2.45	13.9

The lysimetric tanks were filled with sandy loams soil (United States Department of Agriculture classification) with the following physical and chemical properties: sand, 84.7%; silt, 3.3%; clay, 12.0%; field capacity (measured by pressure plate apparatus at -0.03 MPa) of 13.2% dry weight (dw); wilting point (measured by pressure plate apparatus at -1.5 MPa) of 7.2% dw, and a bulk density of 1.45 Mg m^-3^; pH 8.3; electrical conductivity, 0.10 dS m^−1^; organic matter, 0.32% (Walkley and Black method); available phosphorus (Olsen method), 35.6 mg kg^-1^; total potassium, 0.92 g kg^-1^ (determined by coupled plasma optical emission spectrometer, Agilent, ICP-OES 720); total nitrogen, 0.51% (Kjeldahl method); mineral NO_3_–N, 0,7 mg kg^−1^; mineral NH_4_-N, 2.7 mg kg^−1^. This type of soil is characteristic of the Ionian-Metapontine coastline and is extensively employed for vegetable cultivation (Candido et al., 2013). Additionally, this soil has allowed us to operate under favorable hydraulic conductivity conditions, enabling the monitoring of the solution’s movement circulating in the soil through the use of moisture sensors. Three plants were transplanted into each tank, and throughout the cultivation cycle, typical agronomic practices for growing and processing tomatoes in Basilicata were followed. Each lysimeter was periodically irrigated using a micro-flow irrigation system, with drippers installed at each plant, during the cultivation cycle. Following the initial irrigation, which was carried out by applying a volume of water sufficient to return the entire volume of soil to the Field Water Capacity (FWC), a weekly irrigation rotation with an irrigation volume suitable for returning the soil moisture to the FWC was carried out (Allen et al., 1998). The crop water consumption between irrigations was calculated by weighing the individual lysimeter tanks with a trans pallet equipped with load cells. The difference in tank weight between the end of the previous irrigation and the start of the next one represents the water consumption during that time interval as well as the irrigation volume required to restore the soil’s FWC. A probe was inserted in each experimental plot’s lysimeter to test the validity of the irrigation scheduling criterion and maybe correct the specific volume of watering. A scanner outfitted with Diviner 2000 sensors from Sentek Technologies was used to monitor soil moisture. We were able to accurately monitor all components of the water balance and collect drainage water samples to trace any EC movement in the aquifer thanks to the lysimeters. In this regard, because the tomato test takes place in a protected setting, irrigation volume was purposely raised at a given moment during the growing cycle to induce drainage.

### 2.2 Emerging contaminants extraction from waters, soils, and plant organs

The concentration of ECs in water samples (spiked wastewaters and leached waters) was evaluated using an online solid phase extraction (SPE) method using previously established analytical settings (UPLC-QTOF/MS/MS) (Montagna et al., 2020). To extract ECs from soils, the modified QuEChERS method (De Mastro et al., 2022b) was used. Before extracting ECs from various parts of the plant, roots were gently hand washed with tap water to remove soil residues, then rinsed with deionized water and blotted dry with a paper towel. Finely chopped roots, leaves, stems, and tomatoes were stored in a 50-mL centrifuge tube in the dark at -20°C until extraction. In a 50 mL plastic centrifuge tube, 2 g of roots, leaves, and stems or 10 g of tomato fruits were placed and spiked with the appropriate recovery surrogate. Except for the tomatoes, 6 mL of water was added to the centrifuge tubes before capping and vortexing for 1 minute. After thoroughly wetting the samples, 10 mL of Acetonitrile was added to the centrifuge tubes and shaken by hand for 5 minutes. After this step, only the leaves, stems, and fruits were allowed to rest for 15 minutes. After that, a salting out step with Citrate buffer (4 g MgSO_4_, 1 g NaCl, 0.5 g NaCitrate dibasic sesquihydrate, 1 g NaCitrate tribasic dihydrate) was performed. For 5 minutes, the tubes were vigorously shaken by hand. Following that, the samples were centrifuged for 5 minutes at 3700 rpm, resulting in a phase separation of the aqueous and organic solvents. The upper ACN layer (6 mL) was transferred into 15 mL tubes for the clean-up step. Tubes containing 900 mg MgSO_4 + _150 mg primary secondary amine (PSA) for roots, 900 mg MgSO_4 + _150 mg PSA + 150 mg octadecyl (C18) for leaves and stems, 900 mg MgSO150 mg PSA + 15 mg graphitized carbon black (GCB) for fruits, were vortexed for 1 min. After centrifugation (5 min, 4000 rpm), the supernatant was filtered through a membrane filter (PVDF, 0.22 μm), and 1.5 mL was transferred into a screw cap vial for LC-MS/MS analysis to determine the concentration of ECs from the four replicates of each thesis.

### 2.3 Statistical analysis

The ANOVA procedure was applied to all datasets using a randomized complete design with four replicates. A one-way ANOVA procedure (Christensen, 2020) was used with the irrigation typology (FW, TWWx1, and TWWx3) as fixed factors and the replication as random. The entire dataset was tested using the analysis of variance (ANOVA) assumptions. The normality distribution of the model’s residuals was verified graphically (QQ-plot) and statistically (Shapiro-Wilk normality test). Furthermore, Levene’s test was used to confirm homoscedasticity. The experimental design and random sampling for the different matrices met the final ANOVA assumption. When all three ANOVA assumptions were met, the ANOVA was applied to the model. Only when the ANOVA revealed a significant difference (p-value 0.05), was a *post hoc* analysis of the estimated marginal averages performed using Tukey’s HSD (honestly significant difference) test from the R package agricolae (de Mendiburu and Yaseen, 2020).

## 3 Results

### 3.1 Water balance components

**Figure 4:**
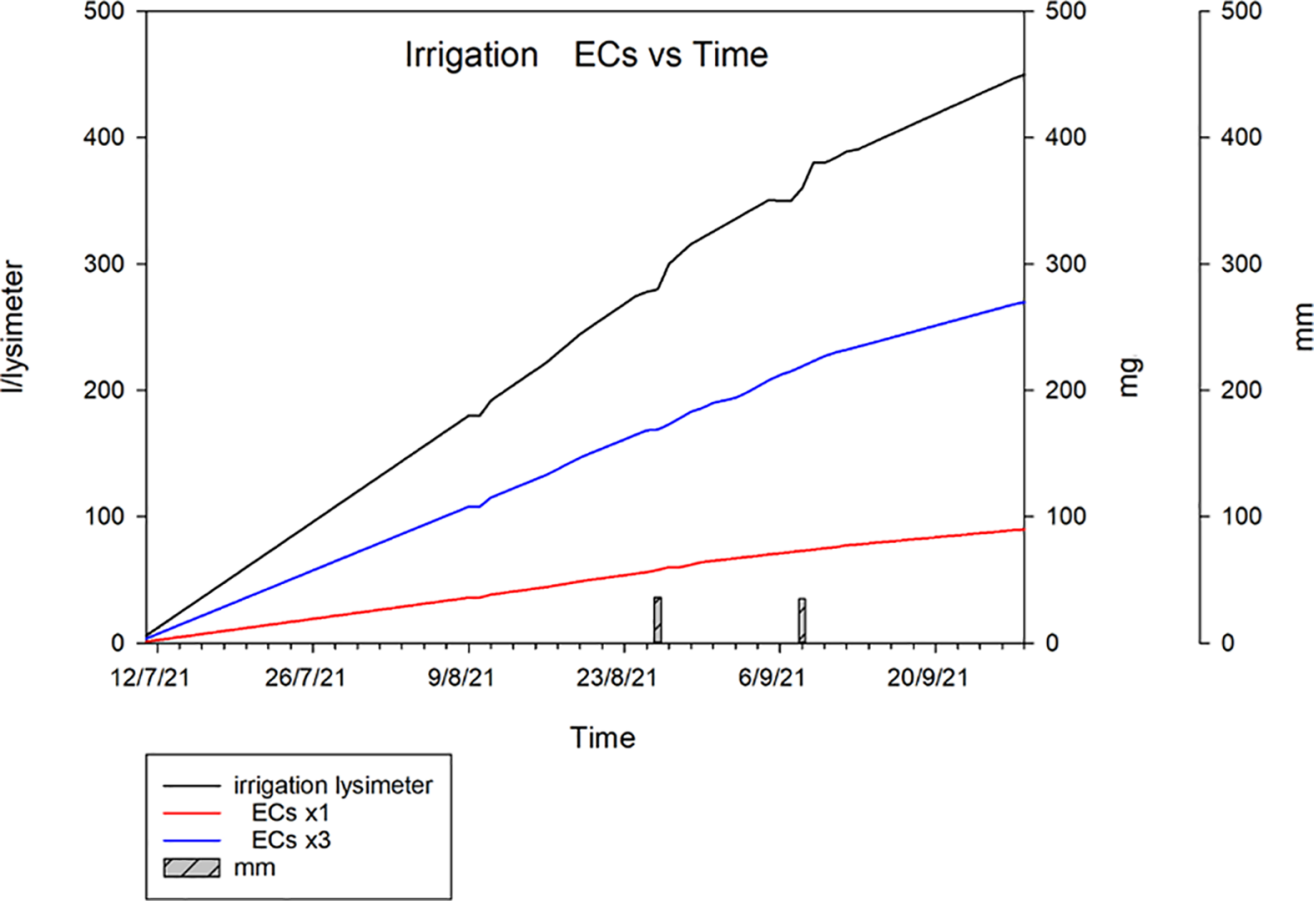
Cumulative water and ECs applied to the soil using fresh water (FW) and TWW effluent spiked with the addition of target contaminants in a dose comparable to the European average (TWWx1) and a triple dose (TWWx3).

**Table 2** depicts the main components of water balance (seasonal irrigation volume, rainfall, and drainage), as well as the total ECs intake in the lysimeters. The total amount of applied irrigation water (I.V.) was 620.8 mm, while the total amount of drained water (D) was 25 mm. The total rainfall for the tomato growth cycle (R)was 88 mm. **Figure 4** depicts the total amount of water and ECs applied to the soil using the TWWx1 and TWWx3 irrigation treatments.

**Table 2:** The volume of seasonal irrigation, total rainfall, drainage, and ECs intake during the tomato growing cycle.

Parameter	Unit	FW	TWWx1	TWWx3
Total rainfall during the tomato growing cycle (R)	mm	88	88	88
Seasonal irrigation volume (I.V.)	mm	620.8	620.8	620.8
Total amount of drained water (D)	mm	25.0	25.0	25.0
Irrigation on lysimeter (time of flowering)	L lysimeter^-1^	250	250	250
Total ECs intake in lysimeters (time of flowering)	mg	0	50	150
Irrigation on lysimeter (end season)	L lysimeter^-1^	449.7	449.7	449.7
Total ECs intake in lysimeters (end season)	mg	0	89.94	269.82

### 3.2 Concentration, accumulation, and fate of ECs

**Figure 5:**
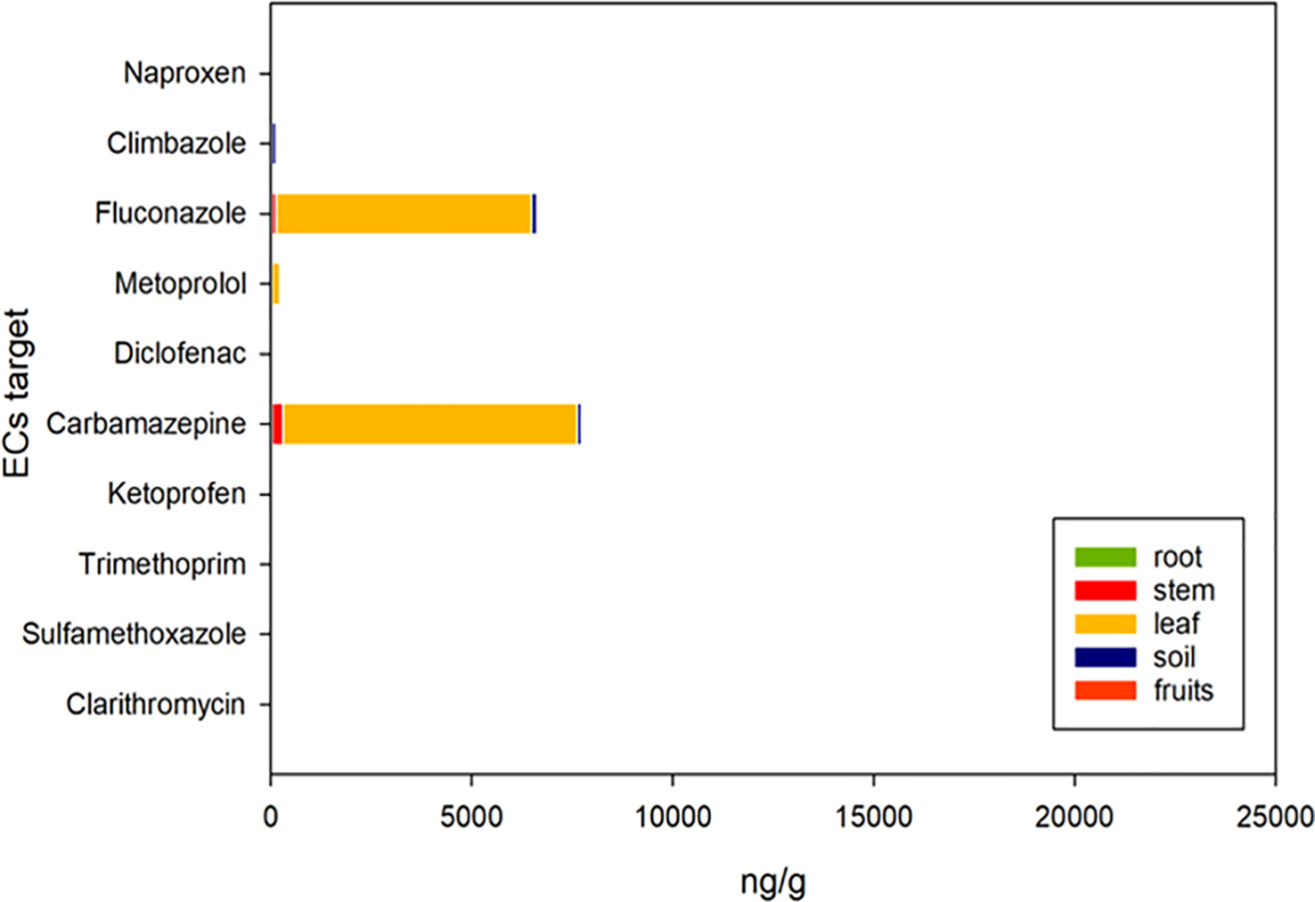
Cumulative concentrations of ECs in the plant-soil environment using fresh water (FW) and TWW effluent (TWWx1).

**Figure 6:**
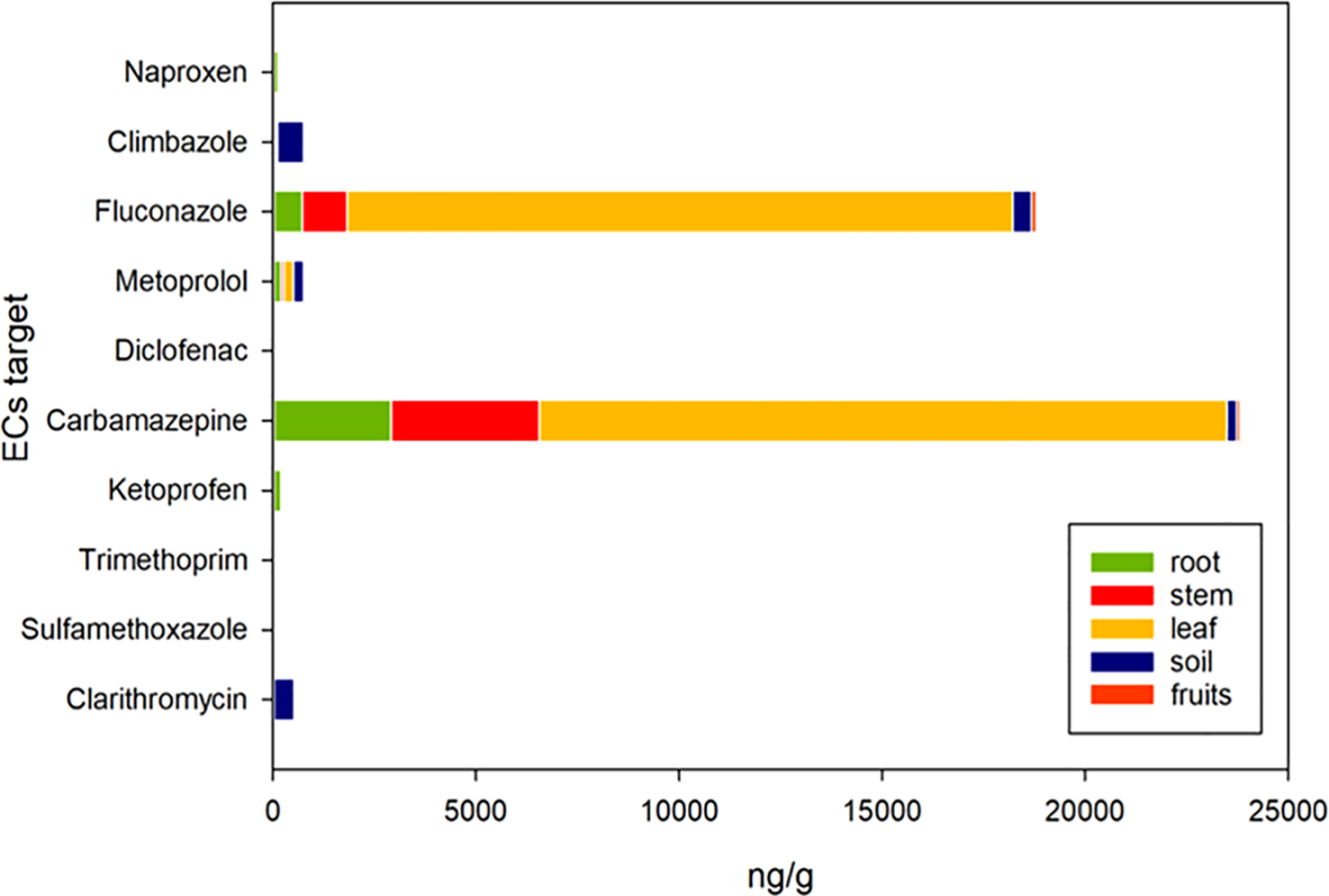
Cumulative concentrations of ECs in the plant-soil environment using fresh water (FW) and TWW effluent (TWWx3).

**Figures 5** and **6** depict the final concentrations of ECs in soil and plant matrices (root, stem, leaf, and fruits) at the end of the cultivation cycle. The FW irrigation approach contained no significant concentrations of target ECs. The TWWx1 method acted differently for each matrix (**Figure 5**). Rather than the fruit, the leaves had high levels of two ECs, fluconazole, and carbamazepine. The residual pollutant amounts in plant tissues were not substantially different from zero. Fluconazole, carbamazepine, and metoprolol levels in plant leaves, roots, stems, and fruits increased significantly with the TWWx3 strategy (**Figure 6**). The concentrations of the remaining contaminants in plant tissues were not significantly different from zero. The largest quantities of the three pollutants observed in the plants (fluconazole, carbamazepine, and metoprolol) were found in the leaves in both irrigation treatments (TWWx1 and TWWx3), with lower but substantial concentrations reported in the stems, roots, and fruits. Martínez-Piernas et al., 2019 observed similar results, where organic microcontaminant concentrations were lower in tomato fruits, generally 10 times lower in fruit compared to leaves. Significant quantities of climbazole, fluconazole, carbamazepine, sulfamethoxazole, and clarithromycin were discovered in soil irrigated with TWWx1 and TWWx3 water. The other five pollutants in the soil had statistically negligible concentrations (**Figures 5**, **6**). Results of Pico et al. (2019) study revealed the potential uptake and accumulation by crops of carbamazepine (as 10,11-carbazepine epoxide), atenlolol, caffeine, gemfibrozil and ibuprofen (as ibuprofen hexoside). Some pharmaceuticals and seven pesticides were detected in plants. Pharmaceuticals and ECs were found in quantifiable levels in all irrigation water, soils, and plants (>99.6%) in Israel (Ben Mordechay et al., 2022). Martínez-Piernas et al. (2019) revealed the presence of 17 OMCs in leaves and 8 in fruits with a higher frequency of detection of carbamazepine, evidencing their higher capability of uptake and translocation within the plant. Sunyer-Caldú et al. (2023) found that pharmaceuticals were the most frequently detected ECs in soils and waters, whereas UV filters achieved the highest concentrations. Diclofenac and salicylic acid were the most accumulated in soils, and diclofenac, ofloxacin, and benzophenone-4 were the most prevalent in the WWTP effluent. Camacho-Arévalo et al. (2021) analyzing the fate of sulfonamide antibiotics in tomato crops in commercial greenhouses in Almería (Spain) found that sulfamethoxazole was the antibiotic with the highest concentration in tomato fruit and irrigated soils. Christou et al. (2017) in a long-term (three consecutive years) wastewater irrigation of a tomato crop found that the highest soil concentration was due to sulfamethoxazole whereas diclofenac displayed the highest fruit concentration. The concentration of the studied pharmaceuticals in both the soil and tomato fruits varied depending on the qualitative characteristics of the treated effluent applied and the duration of WW irrigation. EC concentrations in irrigation water, as well as their physiochemical properties (primarily charge and lipophilicity), are the primary determinants of their translocation and accumulation in the soil-plant continuum (Ben Mordechay et al., 2022).

### 3.3 Mass balance of the ECs

The mass balance of the 10 ECs presented in this study was computed using the lysimetric technique utilized in this investigation for the soil, plant, and water compartments. **Tables 3** and **4** indicate the total ECs intake in the systems (lysimeter) via irrigation water (90 and 270 mg lysimeter^-1^ of each EC, respectively, plus the amount present in the freshwater); the same tables also show the number of ECs detected in plants, leached water, and soil in the TWWx1 and TWWx3 treatments. The not detected column is the residue of the mass balance between ECs intake and the measured sum of ECs accumulated in plants, leached water, and soil.

**Table 3:** Total intake and EC accumulation in plants, leached water, and soil lysimeters in the TWWx1 treatment (mean values of three replicates are shown).

Late season TWWx1 (mg lysimeter^-1^)					
Target	Plant	Leached water	Soil	not detected	Total ECs intake
Clarithromycin ***	0 c	0 c	41.99 b	47.95 a	90
			47%	53%	
Carbamazepine ***	2.41 b	0.83 b	44.13 a	42.63 a	90
	3%	1%	49%	47%	
Fluconazole***	2.00 d	12.90 c	51.87 a	23.17 b	90
	2%	14%	58%	26%	
Climbazole ***	0 b	0 b	90.00 a	0 b	90
			100%		
Sulfamethoxazole ***	0 c	2.86 b	3.18 b	83.90 a	90
		3%	4%	93%	
Trimethoprim ***	0 c	0 c	11.61 b	78.30 a	90
			13%	87%	
Ketoprofen ***	0 b	0 b	0 b	89.94 a	90
				100%	
Diclofenac ***	0 b	0 b	0 b	89.98 a	90
				100%	
Metoprolol ***	0.07 c	0 c	9.72 b	80.15 a	90
			11%	89%	
Naproxen ***	0 b	0 b	0 b	89.94 a	90
				100%	

Different letters and * indicate statistical differences among different theses (p < 0.05). p < 0.05 (*), p < 0.05 (**), p < 0.001 (***), ns (non-significant).

The column not detected is calculated as a residual of the mass balance of each experimental treatment. The percentage of each voice of the mass balance is calculated with respect to the total ECs intake.

**Table 4:** Total intake and EC accumulation in plants, leached water, and soil lysimeters in the TWWx1 treatment (mean values of three replicates are shown).

Late season TWWx3 (mg lysimeter^-1^)					
Target	Plant	Leached water	Soil	not detected	Total ECs intake
Clarithromycin ***	0 c	0 c	202.44 a	67.38 b	270
			75%	25%	
Carbamazepine ***	10.03 c	11.12 c	104.65 b	145.02 a	270
	4.50%	4%	39%	53%	
Fluconazole***	6.37 d	44.68 b	189.24 a	30.1 c	270
	2.50%	17%	70%	11%	
Climbazole ***	0.9 c	0 d	245.17 a	24.56 b	270
			91%	9%	
Sulfamethoxazole ***	0 c	21.53 b	15.38 b	232.87 a	270
		8%	6%	86%	
Trimethoprim ***	0 c	0 c	44.23 b	225.77 a	270
			16%	84%	
Ketoprofen ***	0 c	0 c	2.25 b	267.4a	270
			1%	99%	
Diclofenac ***	0.04 b	1.64 b	O b	268.14 a	270
		1%		99%	
Metoprolol ***	0.27 c	0 c	83.23 b	186.31 a	270
			31%	69%	
Naproxen ***	0.4 b	0 c	0 c	269.71 a	270
				100%	

Different letters and * indicate statistical differences among different theses (p < 0.05). p < 0.05 (*), p < 0.05 (**), p < 0.001 (***), ns (non-significant).

The column not detected is calculated as a residual of the mass balance of each experimental treatment. The percentage of each voice of the mass balance is calculated with respect to the total ECs intake.

According to the mass balance, no ECs were found in the FW treatment; however, contaminants accumulation in the soil-plant-water system was measured for some ECs in the TWWx 1 (**Table 3**) and TWWx 3 (**Table 4**) treatments, with varying behavior among the ECs. Naproxen and diclofenac were not found in the plant tissues, soil, or drainage water of any of the irrigation treatments (**Tables 3**, **4**). This means that nearly all of these ECs are degraded in different chemical by-products. Ketoprofen behaved similarly to naproxen and diclofenac, except for a 1% accumulation in the soil in the TWWx3 treatment (**Table 4**).

Climbazole, clarithromycin, trimethoprim, metoprolol, and sulfamethoxazole accumulated in the soil as a percentage of the total amount of irrigation added to the system, with values ranging from 100%, 47%, 13%, 11%, and 4% in TWWx1 to 91%, 75%, 16%, 31%, and 6% in TWWx3 (**Table 3**, **4**). Except for climbazole (1% in TWWx3) and sulfamethoxazole (3% and 8% in TWWx1 and TWWx3) in drainage water, no accumulation of these five ECs was detected in plant tissues or leached water. We assume that naproxen and diclofenac were degraded in by-products because the residual amount of these five ECs concerning total intake was not detected.

Fluconazole and carbamazepine were found in the soil, plant tissues, and drainage water. Carbamazepine accumulated in plant tissues, drainage water, and soil at a rate of 3%, 1%, and 49% of the total amount added to the system with irrigation in TWWx1 and 4,5%, 4%, and 39% in TWWx3. The balance that was not detected (47% and 53% in TWWx1 and TWWx3) is assumed to be degraded in by-products (**Tables 3**, **4**). Fluconazole accumulated in plant tissues, drainage water, and soil at rates of 2%, 14%, and 58% in TWWx1 and 2.5%, 17%, and 70% in TWWx3. The balance’s undetected residual (26% in TWWx1 and 11% in TWWx3, respectively) is assumed to be degraded in by-products (**Tables 3**, **4**).

### 3.4 The concentration of EC on tomato fruit

**Table 5** shows the average EC concentrations in tomato fruits. All fruits’ concentrations are given in fresh weight, with a ripe tomato containing 95% water and 5% dry matter. The results showed that the contaminants under study had varying concentrations and behaviors. None of the ten contaminants evaluated were discovered in significant concentrations in FW or TWWx1-irrigated tomatoes (**Table 4**, **Figure 5**). Some contaminants responded differently after TWWx3 treatment (**Table 4**, **Figure 6**). During the TWWx3 strategy, only fluconazole, carbamazepine, metoprolol, clarithromycin, climbazole, and sulfamethoxazole were identified in fruits. The concentrations of the individual compounds varied significantly: fluconazole was 110 ng g^-1^, carbamazepine was 89.2 ng g^-1^ and metoprolol was 1.22 ng g^-1^. Clarithromycin, climbazole, and sulfamethoxazole were found at 0.03 ng g^-1^ concentrations, which was statistically comparable to 0. Christou et al. (2017) discovered that diclofenac, sulfamethoxazole, and trimethoprim concentrations in soil were 0.35, 0.98, and 0.62 μg kg^−1^, respectively. For fruit, diclofenac, sulfamethoxazole, and, trimethoprim concentrations were 11.63, 5.26, and 3.4 μg kg^−1^, respectively. The average carbamazepine content in tomato leaves was 8.9 ng g^−1^ while in fruit was 0.23 ng g^−1^ (Martínez-Piernas et al., 2019). In tomato mature plants grown on fortified water-irrigated plots, the concentration of carbamazepine was found to be 0.19 ± 0.32 ng g^-1^ (Wu et al., 2014). Ben Mordechay et al. (2022) discovered that the average EC content in soils was 129.4 88.5 g ha^-1^, whereas the concentration of carbamazepine on tomato leaves was 546.4 557.5 ng g^-1^.

**Table 5:** Tomato fruits ECe concentrations and ECe leachate total amount in the three irrigation treatments (mean values of three replicates are shown).

ECs target	Fruits			Leachate		
	TWWx1 (ng g^-1^)	TWWx3 (ng g^-1^)	FW (ng g^-1^)	TWWx1 (mg lysimeter ^-1^)	TWWx3 (mg lysimeter ^-1^)	FW (mg lysimeter ^-1^)
Fluconazole	–	110 a	–	11.9 a	44.6 a	–
Carbamazepine	–	89.2 b	–	2.9 b	21.2 b	–
Metoprolol	–	1.2 c	–	0.9 c	11 c	–
Clarithromycin	–	0.4 d	–	0 d	1.5 d	–
Climbazole	–	0.3 d	–	0 d	0 e	–
Sulfamethoxazole	–	0.3 d	–	0 d	0 e	–
Diclofenac	–	0 d	–	0 d	0 e	–
Ketoprofen	–	0 d	–	0 d	0 e	–
Naproxen	–	0 d	–	0 d	0 e	–
Trimethoprim	–	0 d	–	0 d	0 e	–
*Signif. codes*	ns	***	ns	**	***	ns

## 4 Discussion

The European summers of 2018, 2019, and 2020 caused widespread and severe droughts, setting a new standard in Europe (Rakovec et al., 2022). Given the increasing scarcity and pressure on freshwater resources for irrigation, the use of alternative water resources such as treated wastewater is becoming more popular. The use of treated wastewater as a potential source of fresh water is expected to gain popularity not only in arid regions but also in temperate climates (Hochstrat et al., 2006). However, it should be noted that (unregulated) de facto (indirect) reuse has been common practice for decades (Beard et al., 2019). A new EU regulatory framework now intends to stimulate and regulate the direct reuse of treated domestic wastewater for irrigation purposes (EU). Because responsible reuse is critical (Dingemans et al., 2020) a risk management plan is part of the EU regulation 2020/74, which includes the effect of water reuse on farmers, soil, groundwater, and ecosystems. However, there is currently no direct data on the effects of reusing treated wastewater irrigation under real-world agricultural conditions on the fate of a diverse variety of ECs (Narain-Ford et al., 2022). To date, only a few studies have shown that crop plants irrigated with treated wastewater in the field or in simulated field settings absorb and accumulate emerging contaminants. Quantifying the ECs investigated in the plant-soil environment is critical because it will provide a better understanding of crop plants’ ability to absorb and accumulate ECs. In this study, we used a controlled lysimeter experiment to determine the fate of ECs in the soil-water-plant system. According to the findings of the current study, the fate of ECs in the soil-plant water system varies depending on the contaminant. Except for a very minor concentration of ketoprofen in soil irrigated with a triple dose of ECs, the total amount of naproxen, diclofenac, and ketoprofen delivered in lysimeters with irrigation water was not discovered in plant tissues, soil, or drainage water. This implies that 100% of these two ECs are rapidly degraded into by-products with distinct chemical compositions. The formation of by-products that are not necessarily less toxic than the starting compounds is a critical point that needs to be investigated further. Despite extensive research on ECs, little is known about the incidence and destiny of their by-products or metabolites in the environment. At the end of the growing cycle, climbazole, clarithromycin, trimethoprim, metoprolol, and sulfamethoxazole were found in the soil, but no accumulation was found in plant tissues or leached water, with the exception of a small amount of climbazole in plant tissues (1% in TWWx3) and sulfamethoxazole in drainage water (3% and 8% in TWWx1). It should be emphasized that the TWWx3 treatment was used to boost EC concentrations and stress the soil-plant reaction. The presence of clarithromycin, trimethoprim, metoprolol, and sulfamethoxazole in soil but not in plant tissues indicates that either tomato plants have a limited ability to adsorb them or soil particles have a high ability to adsorb them. The ability of the soil to absorb the aforementioned ECs could also explain their lack of drainage water. As with naproxen and diclofenac, climbazole, clarithromycin, trimethoprim, metoprolol, and sulfamethoxazole are assumed to be degraded in by-products in the plant-water system. The time required for degradation may be related to the difference in the percentage of ECs detected versus those not detected in the soil. In TWWx1, for example, metoprolol accumulation was recorded at 11% in the soil and 89% was not identified, implying a faster degradation time than climbazole, which had 100% accumulation in the soil at the same sampling time (**Table 3**). Carbamazepine and fluconazole were found in plant tissues, soil, and drainage water, and they were the least degraded ECs found in by-products. These data show that these two ECs are more persistent in the soil-water system and have a longer degradation period than the other ECs studied. Among the azoles, fluconazole, due to its complex chemical structure, comprising two triazoles and two chlorine atoms, is considered a persistent compound, unlike climbazole and sulfamethoxazole (Pacholak et al., 2022). Other research studies (Christou et al., 2017; Martínez-Piernas et al., 2019; Camacho-Arévalo et al., 2021; Sunyer-Caldú et al., 2023) have demonstrated that other contaminants such as diclofenac and sulfamethoxazole remain a concern. Carbamazepine is one of the most frequently detected ECs in soils irrigated with reclaimed water (Beltrán et al., 2020), and these findings suggest that these contaminants have a high potential for soil and water pollution. The results indicate also an uptake of carbamazepine and fluconazole by plants, as also reported by (De Mastro et al., 2023). In particular, the highest concentrations of the last two contaminants were found in leaf tissues, and only when we forced the ECs concentration in the TWWx3 treatment were carbamazepine and fluconazole found in fruit tissues. Most studies that found the absence of most added compounds in tomato fruits can be explained by increased water flow for transpiration towards the leaves, resulting in a greater accumulation of ECs in the leaves than in the fruits, as demonstrated by (Martínez-Piernas et al., 2019). Second, ECs taken up by the plant can be converted into phase I metabolites (for example, hydroxylation) and phase II metabolites, for example, by conjugating the progenitor chemical or phase II metabolites with glucose, glucuronic acid, and malonic acid (Mlynek et al., 2021). Our findings are supported by the metabolization of progenitor components, such as the absence of substances within the fruit, which is consistent with Kovačič et al. (2023). The lack of all of the examined ECs when tomato fruits received irrigation with water containing the average European pollutants concentration appears to imply that the reuse of treated wastewater might be considered a reliable water supply (Kovačič et al., 2023). However, the presence of carbamazepine and fluconazole in plant tissues (roots, stems, and leaves in TWWx1, and fruits in TWWx3) in our study suggests that these two contaminants may be taken up and accumulated in the edible part of the tomato, posing a risk to human health and the food chain. Fruit contamination is possible at high ECs concentrations in irrigation water for Metoprolol (1,2 ng g^-1^ F.W.) and, at very low concentrations, for clarithromycin and sulfamethoxazole. (Bigott et al., 2022) and (Gallego et al., 2021) discovered a trend of higher concentrations of carbamazepine and climbazole in crops irrigated with treated wastewater.

To date, about 90% of emerging contaminants are disposed unscientifically into water bodies, creating problems to public health and environment. Their mitigation remains mainly limited by economic factors. Analysis is also very time consuming and costly and requires access to highly sophisticated equipment. Tarpani and Azapagic (2018) found that the life cycle for advanced effluent treatment range from 0.112 £ m^-3^ for ozonation based to 0.238 £ m^-3^ the highest for solar-Fenton processes. They concluded that advanced wastewater and sludge treatment would increase the costs of conventional wastewater treatment by 1.5–2.1 times. Pryce et al. (2022) analyzing the cost-effectiveness of graphene-based materials (GBMs) for EC removal found that the life cycle cost was 1.73 ± 0.09 $ m^-3^ for graphene-oxide foam adsorbent, 2.97 ± 0.15 $ m^-3^ for porous graphene adsorbent and 2.12 ± 0.11 $ m^-3^ for a hybrid filter. Studies on the economics of advanced wastewater for removing EC are generally limited. As a result, more research is required to understand the long-term consequences on soil quality, crop productivity, and food safety, as well as a cost-benefit analysis of EC removal.

## 5 Conclusions

The effects of treated wastewater on fruit production, specifically tomato production, were investigated in this study. The behavior of various target ECs in the plant-soil complex was studied and found to vary. Fluconazole and carbamazepine, in particular, were shown to have high plant absorption concentrations, with accumulation evident in the leaves, roots, and berries of the TWWx3 treatment. This imply that these two contaminants may be taken up and accumulated in the edible part of the tomato, posing a risk to human health and the food chain. However, other ECs (such as sulfamethoxazole, trimethoprim, ketoprofen, diclofenac, metoprolol, and naproxen) showed substantial uncertainties in their fate, which was most likely owing to degradation in the soil and cultivation factors. The study’s findings support the premise that constant and proper monitoring of the quality of water used for crop irrigation is necessary to minimize economic and food-quality losses. When properly monitored, reusing treated wastewater for irrigation can be a safe approach in agriculture, and can help policymakers develop future legislative frameworks for sustainable water management. Wastewater reuse adheres to the circular economy principles applied to water management because it can relieve pressure on surface and groundwater resources, provide a more consistent supply of water that is less dependent on climatic variations, and supplement existing water sources. More research on the environmental and health implications of ECs in agricultural systems is required, particularly the creation of metabolites and transformation products, to provide a conclusive answer on the safety of treated wastewater for irrigation.

## Data availability statement

The original contributions presented in the study are included in the article/supplementary material. Further inquiries can be directed to the corresponding authors.

## Author contributions

Conceptualization: MD, VC and MP. Methodology: MD, VC, GG, MG and MP. Data curation: MD and AM. Formal analysis: MD and MP. Resources: MD, VC, GB, FD, SM, CS, CD and MP. Investigation: MD, VC, AM and MP. Writing - original draft: MD and AM. Writing - review & editing: MD, VC, GB, FD, SM, CS, CD, GG, MG, RB, AM, MP. Supervision and project administration: VC and MP. All authors contributed to the article and approved the submitted version.
